# Poor Agreement between Pulmonary Capillary Wedge Pressure and Left Ventricular End-Diastolic Pressure in a Veteran Population

**DOI:** 10.1371/journal.pone.0087304

**Published:** 2014-01-31

**Authors:** Abbas Bitar, Mona Selej, Islam Bolad, Tim Lahm

**Affiliations:** 1 Department of Medicine, Internal Medicine Residency Program, Krannert Institute of Cardiology, Indiana University, Indianapolis, Indiana, United States of America; 2 Department of Medicine, Division of Pulmonary, Allergy, Critical Care, Occupational and Sleep Medicine, Krannert Institute of Cardiology, Indiana University, Indianapolis, Indiana, United States of America; 3 Department of Medicine, Krannert Institute of Cardiology, Indiana University, Indianapolis, Indiana, United States of America; 4 Richard L. Roudebush Veterans Affairs Medical Center, Indianapolis, Indiana, United States of America; Virginia Commonwealth University, United States of America

## Abstract

**Background:**

Accurate determination of left ventricular filling pressure is essential for differentiation of pre-capillary pulmonary hypertension (PH) from pulmonary venous hypertension (PVH). Previous data suggest only a poor correlation between left ventricular end-diastolic pressure (LVEDP) and its commonly used surrogate, the pulmonary capillary wedge pressure (PCWP). However, no data exist on the diagnostic accuracy of PCWP in veterans. Furthermore, the effects of age and comorbidities on the PCWP-LVEDP relationship remain unknown.

**Methods:**

We investigated the PCWP-LVEDP relationship in 101 patients undergoing simultaneous right and left heart catherization at a large VA hospital. PCWP performance was evaluated using correlation and Bland-Altman analyses. Area under Receiver Operating Characteristics curves (AUROC) for PCWP were determined.

**Results:**

PCWP-LVEDP correlation was moderate (r = 0.57). PCWP-LVEDP calibration was poor (Bland-Altman limits of agreement −17.2 to 11.4 mmHg; mean bias −2.87 mmHg). 59 patients (58.4%) had pulmonary hypertension; 15 (25.4%) of those met pre-capillary PH criteria based on PCWP. However, if LVEDP was used instead of PCWP, 7/15 patients (46.6%) met criteria for PVH rather than pre-capillary PH. When restricting analysis to patients with a mean pulmonary artery pressure of ≥25 mmHg and pulmonary vascular resistance of >3 Wood units (n = 22), 10 patients (45.4%) were classified as pre-capillary PH based on PCWP ≤15 mmHg. However, if LVEDP was used, 4/10 patients (40%) were reclassified as PVH. Among patients with any type of pulmonary hypertension, PCWP discriminated moderately between high and normal LVEDP (AUROC, 0.81; 95%CI 0.69–0.94). PCWP-LVEDP correlation was particularly poor in patients with COPD or obesity.

**Conclusion:**

Reliance on PCWP rather than LVEDP results in misclassification of veterans as having pre-capillary PH rather than PVH in almost 50% of cases. This is clinically relevant, as misclassification may lead to inappropriate therapies and adverse events.

## Introduction

Pulmonary hypertension (PH) is present when the mean PA pressure (mPAP) is ≥25 mmHg [1], [2]. A diagnosis of pulmonary arterial hypertension (PAH), the only PH subtype for which specific pulmonary vasodilators are FDA-approved, requires absence of left ventricular (LV) dysfunction [3]. The management of PH with elevated LV filling pressures (WHO group II PH; also referred to as “post-capillary PH”, “pulmonary venous hypertension” or “PVH”) differs dramatically from pre-capillary PH, which encompasses PAH and other PH forms with normal LV pressures [4], [5]. In fact, the pulmonary vasodilators used for PAH worsen morbidity and mortality in PVH [4]–[7]. Right heart catheterization (RHC) with accurate assessment of LV filling pressures is therefore of utmost importance in making a correct diagnosis of PAH, in selecting appropriate therapies, and in differentiating PVH from pre-capillary PH.

LV filling pressures can be measured *directly* via left heart catherization (LHC) and assessment of LV end-diastolic pressure (LVEDP), or *indirectly* by measuring pulmonary capillary wedge pressure (PCWP) during RHC. However, a recent study suggested that when relying on measurement of PCWP alone, patients get misclassified (assigned to the wrong WHO PH group) in 50% of cases [8]. This is due to poor correlation and poor agreement between PCWP and the more accurate (but less frequently measured) LVEDP. Whether age and comorbidities affect PCWP-LVEDP relationship remains unknown.

Furthermore, no data exist on the diagnostic accuracy of PCWP in the veteran population. The Veterans Health Administration (VHA) is the largest integrated health care system in the USA and serves 8.3 million veterans annually. Patients are characterized by male predominance, advanced age, and a large burden of chronic cardiopulmonary diseases [9]. For example, the number of veterans ≥85 years treated by the VHA has tripled from 2000 to 2011. Since cardiopulmonary hemodynamics are affected by age and gender [10]–[12], and since veterans are older and exhibit different male-female ratios than previously studied populations [8], [13], data from other populations cannot be extrapolated to VA patients. Specific data for the veteran population are therefore needed. Furthermore, given the large prevalence and prognostic significance of PH in the veteran population [14], it is important to test whether the hemodynamic values obtained in this population are accurate.

Since misclassification of veterans with PVH as pre-capillary PH would be a particular problem in this population with poor cardiopulmonary reserve [9], we sought to determine the correlation between PCWP and LVEDP in patients undergoing combined RHC and LHC at a large VA medical center. A secondary aim was to determine how age and comorbidities affect the PCWP-LVEDP relationship.

## Methods

### Patient population

**Figure 1:**
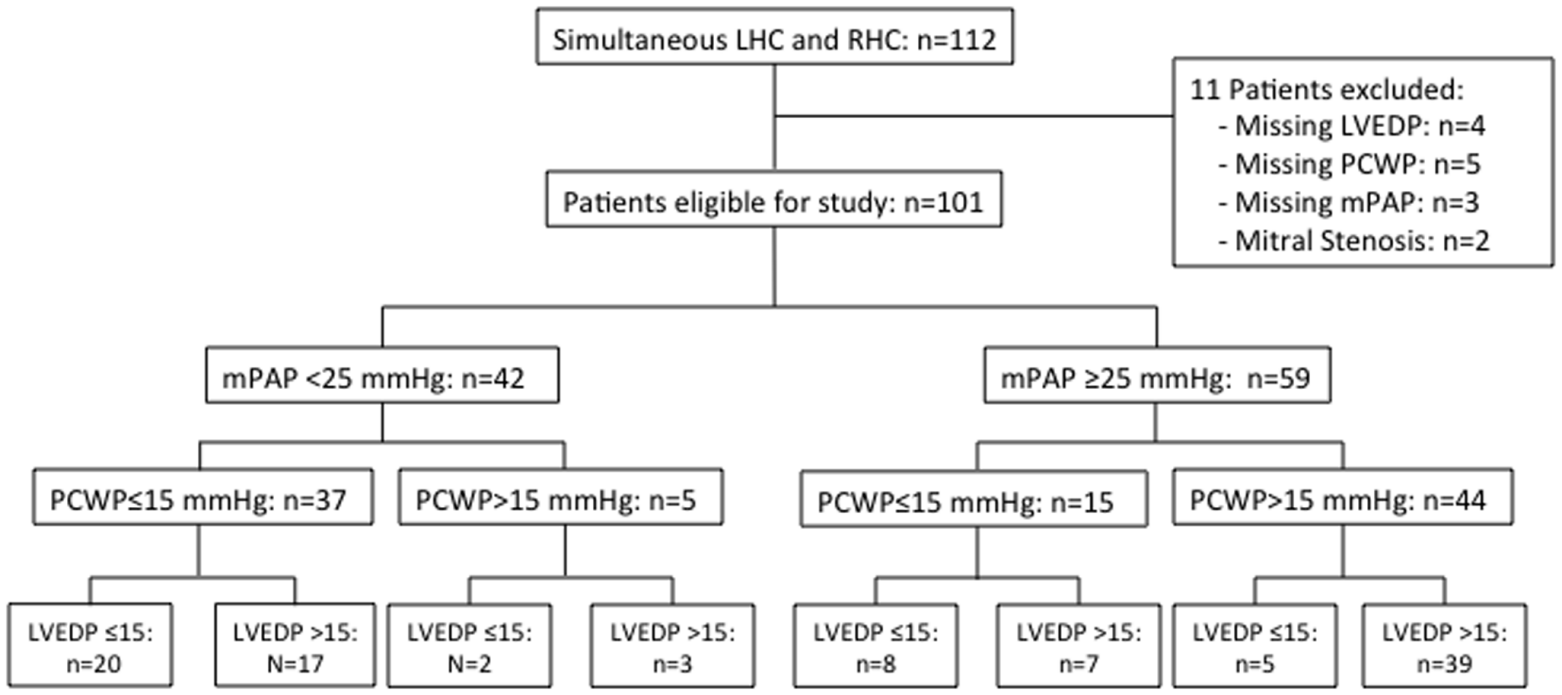
Patient flow chart. LHC, left heart catheterization; LVEDP, left ventricular end-diastolic pressure; mPAP, mean pulmonary artery pressure; PCWP, pulmonary capillary wedge pressure; RHC, right heart catheterization.

We retrospectively analyzed patients ≥18 years that underwent combined RHC and LHC from January 2010 until March 2012 at the Richard L. Roudebush VA Medical Center in Indianapolis, Indiana, USA, a large VA tertiary referral center affiliated with Indiana University School of Medicine. Exclusion criteria included: moderate or severe mitral stenosis, heart rate >130/minute, and missing mPAP, PCWP or LVEDP during RHC/LHC. A total of 112 patients were identified. Eleven patients met exclusion criteria (Figure 1). All information gathered was obtained during routine patient care. This work was approved and granted “exempt” status by the Indiana University Institutional Review Board (IRB; Protocol #: 1201007857). Individual patient consent was waived by the approving IRB.

### Hemodynamic measurements

PH was diagnosed if the mPAP was ≥25 mmHg [1]. Group II PH was diagnosed as PH with PCWP >15 mmHg [1]. All procedures were performed under the supervision of board-certified cardiologists with specialty training in interventional cardiology. All cardiologists were faculty members of the Indiana University School of Medicine. The physicians performing the catheterizations did not follow standardized protocols for measurement of hemodynamic values; however, values from both RHC and LHC were obtained prior to the injection of contrast media for left ventriculography or coronary angiography. Catheters were connected to the transducer after placement of the tip in the right atrium. After opening the transducer to air and placing it at the mid chest position, the transducer was zeroed, and then the valve was turned to close to air and connected to the catheter tip. The cardiologist then reviewed the tracings as the catheter tip was passed to the different chambers of the right heart and the pulmonary vasculature. Measured pressures were accepted only after review of pressure tracings and confirmation of a true representation of the chamber or vascular pressure. Hemodynamic measurements were transferred directly into electronic spreadsheets and stored in an electronic database (VA CART-CL). The computer-generated values of all hemodynamic measurements were reviewed by the cardiologist and used for diagnosis and analysis if no error was detected. Digitized mean PCWP reading were used; no specific respiratory maneuvers were performed during the wedge measurements.

The pulmonary vascular resistance (PVR) was calculated as the transpulmonary gradient (TPG; mPAP minus PCWP) divided by the cardiac output (with the latter being determined by thermodilution; if that was not done, Fick cardiac output was used). PVR >3 Wood units (WU) was considered increased [1], [2]. TPG was considered increased if it was >12 mmHg[1]. Diastolic pressure gradient (DPG) was determined as diastolic PAP minus PCWP, and a value ≥7 mmHg was considered abnormal [15].

### Echocardiography

All transthoracic echocardiograms were performed for routine patient care according to current guidelines by certified technicians and interpreted by a board certified cardiologist. Echocardiograms were reviewed for the presence of mitral stenosis and other valvular heart diseases.

Clinical diagnoses were made based on standard criteria. Pulmonary function testing, laboratory testing, electrocardiography, polysomnography, and blood pressure measurements were performed according to current guidelines as part of routine patient care. Obesity was defined as a body mass index (BMI) ≥30.

### Statistical analysis

Baseline demographic and clinical variables are descriptively summarized. Continuous variables are expressed as mean ± SD. Categorical data are presented as percent frequency. The calibration of PCWP to LVEDP was assessed using Bland-Altman analysis. The area under the receiver operating characteristic curve (AUROC) was calculated to determine the ability of PCWP to discriminate between patients with LVEDP of ≤15 mmHg and those with LVEDP >15 mmHg. Data collection and analysis was performed using SPSS version 17. P<0.05 was considered statistically significant.

## Results

### Patient characteristics

We analyzed data from 101 patients (Fig. 1; table 1). 93% of patients were male; mean age was 65.5±9.4 years. Comorbidities were common (table 1). 59 patients (58.4%) met PH criteria (Fig. 1). Hemodynamic and echocardiographic parameters are shown in table 2 and table 3. 95% of catheterizations were performed by one of two cardiologists.

**Table 1:** Patient characteristics of the 101 study subjects.

**Age (years)**		65.5±9.4
**Sex**	Male	94 (93.1%)
	Female	7 (6.9%)
**Race**	Caucasian	92 (91.1%)
	African American	8 (7.9%)
	Other	1 (1%)
**Height (cm)**		175±9
**Weight (kg)**		100.2±23.0
**Tobacco use (previous or active)**		78 (78.8%)
**BMI**	<18.5	1 (1%)
	18.5–24.9	22 (22%)
	25–29.9	21 (21%)
	30–34.9	28 (28%)
	35–39.9	15 (15%)
	> = 40	13 (13%)
**Comorbidities**	Obstructive sleep apnea	30 (29.7%)
	COPD	21 (20.8%)
	Hypertension	78 (77.2%)
	Coronary artery disease	61 (61.6%)
	Hyperlipidemia	66 (65.3%)
	Diabetes mellitus	46 (45.5%)
	Congestive heart failure	51 (51.5%)
	• Systolic failure	38 (38.4%)
	• Diastolic failure	17 (17.2%)
**Cardiac rhythm**	Sinus	76 (75.2%)
	Atrial fibrillation/flutter	25 (24.8%)
**h/o cardiac surgery**		19 (19%)
	Coronary artery bypass grafting	16 (15.8%)
	Valve replacement	3 (3%)
**Medications**	Aspirin	76 (75.2%)
	ACE-inhibitor/angiotensin receptor-antagonist	61 (60.4%)
	Beta Blocker	66 (65.3%)
	Calcium channel blocker	19 (18.8%)
	Diuretic	57 (56.4%)
	Nitrate	14 (13.9%)
	Statin	67 (66.3%)
	Phosphodiesterase inhibitor	1 (1%)
	Anticoagulation	17 (16.8%)

Age, height, and weight are expressed as means±SD. All other values are expressed as absolute numbers with percent of the total study population in parenthesis.

**Table 2:** Hemodynamic Parameters of the 101 study subjects.

**Heart rate (bpm) (n = 101)**	73±11
**Systolic blood pressure (mmHg) (n = 101)**	121±22
**Diastolic blood pressure (mmHg) (n = 101)**	66±14
**Left ventricular systolic pressure (mmHg) (n = 100)**	135±31
**Left ventricular diastolic pressure (mmHg) (n = 101)**	19±8
**Right atrial pressure (mmHg) (n = 101)**	10±5
**Mean pulmonary artery pressure (mmHg) (n = 101)**	28±10
**<25 mmHg**	42 (41.6%)
**≥25 mmHg**	59 (58.4%)
**Pulmonary capillary wedge pressure (mmHg) (n = 101)**	16±7.4
**≤15 mmHg**	52 (51.5%)
**>15 mmHg**	49 (48.5%)
**Cardiac output (L/min; thermodilution) (n = 51)**	5.0±1.2
**Cardiac output (L/min; Fick) (n = 97)**	5.0±1.3
**Cardiac index (L/min/m^2^; thermodilution) (n = 50)**	2.3±0.54
**Cardiac index (L/min/m^2^; Fick) (n = 95)**	2.3±0.53
**Pulmonary vascular resistance (Wood units) (n = 98)**	2.6±2.0
**Systemic vascular resistance (dynes/sec/cm^−5^) (n = 80)**	1285±485

If a parameter was not obtained in all 101 patients, the number of patients in which this was measured is indicated in parenthesis in the left column. Values are expressed as means±SD, or as absolute numbers with percent of the total study population in parenthesis.

**Table 3:** Echocardiographic parameters.

**Left ventricular ejection fraction (%)**	48.7±15.56
**Left ventricular hypertrophy**	42 (50.6%)
**Left atrial dilatation**	48 (57.1%)
**Right atrial dilatation**	14 (16.5%)
**Left ventricular dilatation**	14 (16.5%)
**Right ventricular dilatation**	11 (13.4%)
**Mitral stenosis**	0 (0%)
**Mitral Regurgitation**	32 (38.1%)
• **Mild**	19 (22.6%)
• **Moderate**	7 (8.3%)
• **Severe**	6 (7.1%)
**Aortic stenosis**	26 (28.9%)
• **Mild**	3 (3.3%)
• **Moderate**	5 (5.6%)
• **Severe**	18 (20%)
**Aortic regurgitation**	14 (16.5%)
**Tricuspid regurgitation**	17 (19.8%)
**Tricuspid stenosis**	0 (0%)
**Left ventricle posterior wall thickness (cm)**	1.2±0.4
**Interventricular septal wall thickness (cm)**	1.2±0.4

Left ventricular ejection fraction, left ventricular posterior wall thickness, and interventricular septal wall thickness are expressed as means±SD. Other values are expressed as absolute numbers with percent of the total study population in parenthesis.

### PCWP and LVEDP relationship in entire cohort

**Figure 2:**
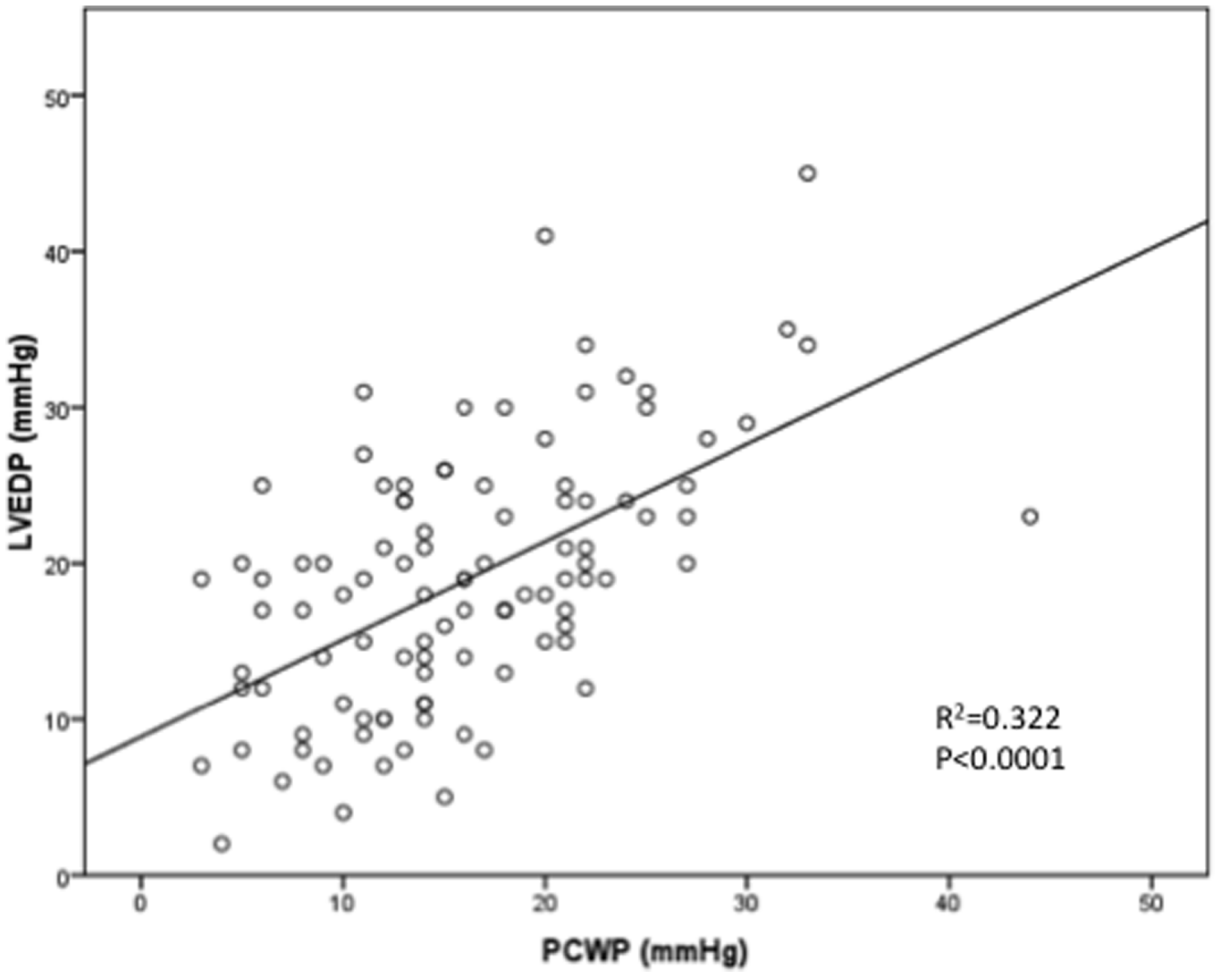
Correlation between PCWP and LEDP in the entire study population. Scatterplot of PCWP and LVEDP pairs for all 101 patients included in the study.

**Figure 3:**
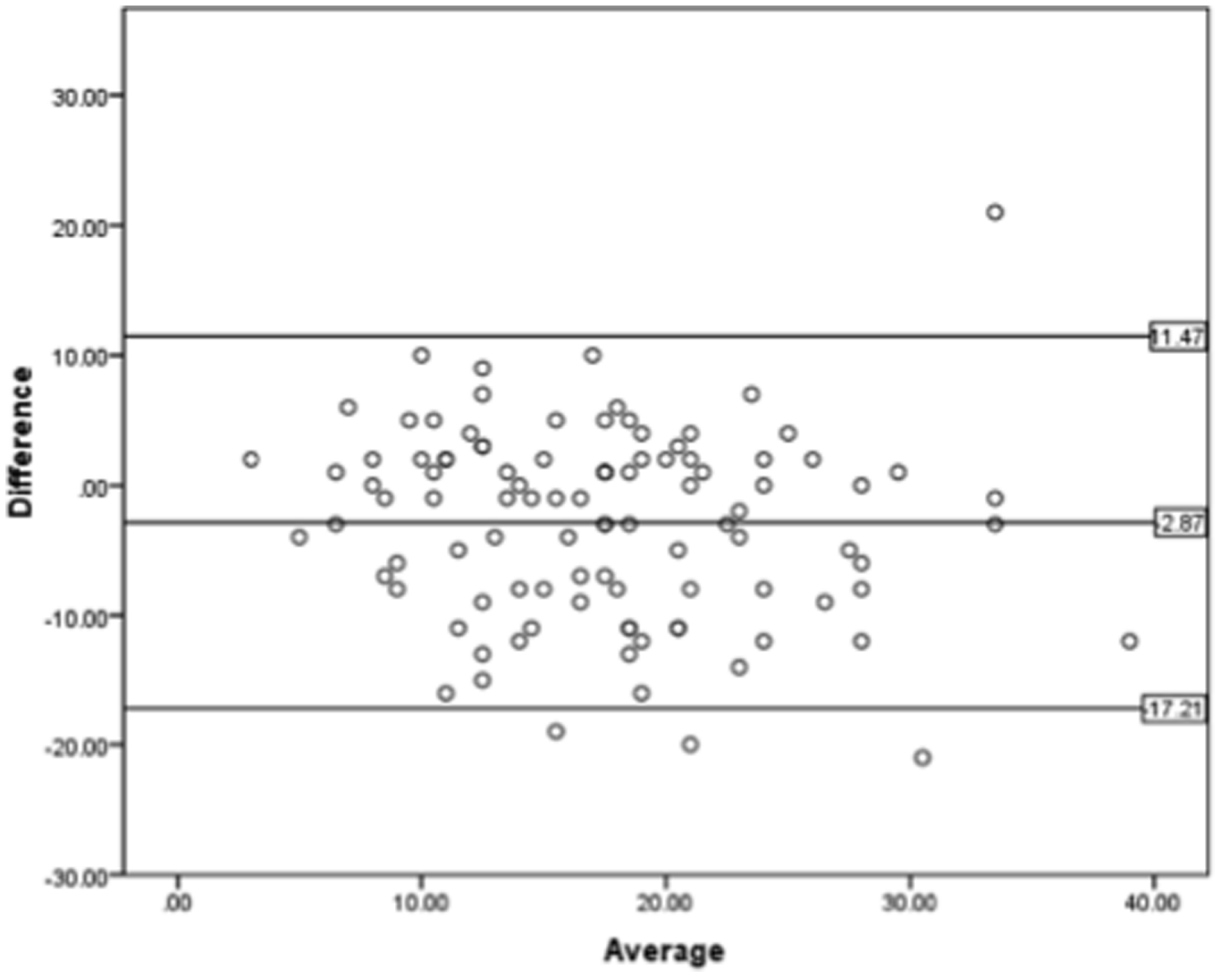
Agreement between PCWP and LEDP in the entire study population. Bland-Altman plot of PCWP and LVEDP pairs for all 101 patients included in the study. Difference indicates difference between PCWP and LVEDP pairs (in mmHg), with positive values indicating that PCWP is higher than corresponding LVEDP for that particular patient, and with negative values indicating that PCWP is lower. Average indicates value of corresponding PCWP and LVEDP pairs divided by 2 ([PCWP+LVEDP/2]). Upper and lower horizontal lines indicate upper and lower borders of 95% limits of agreement, respectively; horizontal line in middle represents mean bias.

PCWP and LVEDP correlation was moderate (r = 0.568, r^2^ = 0.322; Fig. 2). Mean bias was −2.87 mmHg (95%CI −4.316 to −1.42) with 95% limits of agreement ranging from −17.21 to 11.47 mmHg (Fig. 3). This indicates that PCWP underestimates LVEDP on average by 2.87 mmHg, and that even after exclusion of the 5% of patients with the highest differences between PCWP and LVEDP, PCWP underestimated LVEDP by as much as 17.21 mmHg and overestimated it by as much as 11.47 mmHg. In 42.6% of patients the absolute difference between PCWP and LVEDP was >5 mmHg, and in 20.8% of patients >10 mmHg.

Since 18% of our patients were noted to have severe aortic stenosis on echocardiogram (table 3), we performed a sensitivity analysis without these patients. In the remaining 81 patients, the PCWP-LVEDP correlation remained moderate (r = 0.61; r^2^ = 0.376; p<0.001), and the mean bias was −2.65 mmHg (95% CI −4.24 to −1.06), with 95% limits of agreement from −15.90 to 10.60 mmHg. Correlation coefficient and mean bias were thus grossly unchanged compared to the larger cohort that included the patients with severe aortic stenosis.

In order to determine whether there are any specific patient characteristics in those individuals with particularly large discrepancies between PCWP and LVEDP, we identified all patients in which the PCWP-LVEDP difference was >1 standard deviation (SD) from the mean bias (mean bias <−10.15 or >4.43; n = 33). When comparing this cohort with those patients in which the PCWP-LVEDP difference was within 1 SD (mean bias −10.15 to 4.43), we did not find any significant differences in age, sex, BMI, comorbidities, or medications (data not shown), suggesting that there are no particular identifiers of patients with the largest PCWP-LVEDP discrepancies.

### PCWP and LVEDP relationship in patients with or without PH

**Figure 4:**
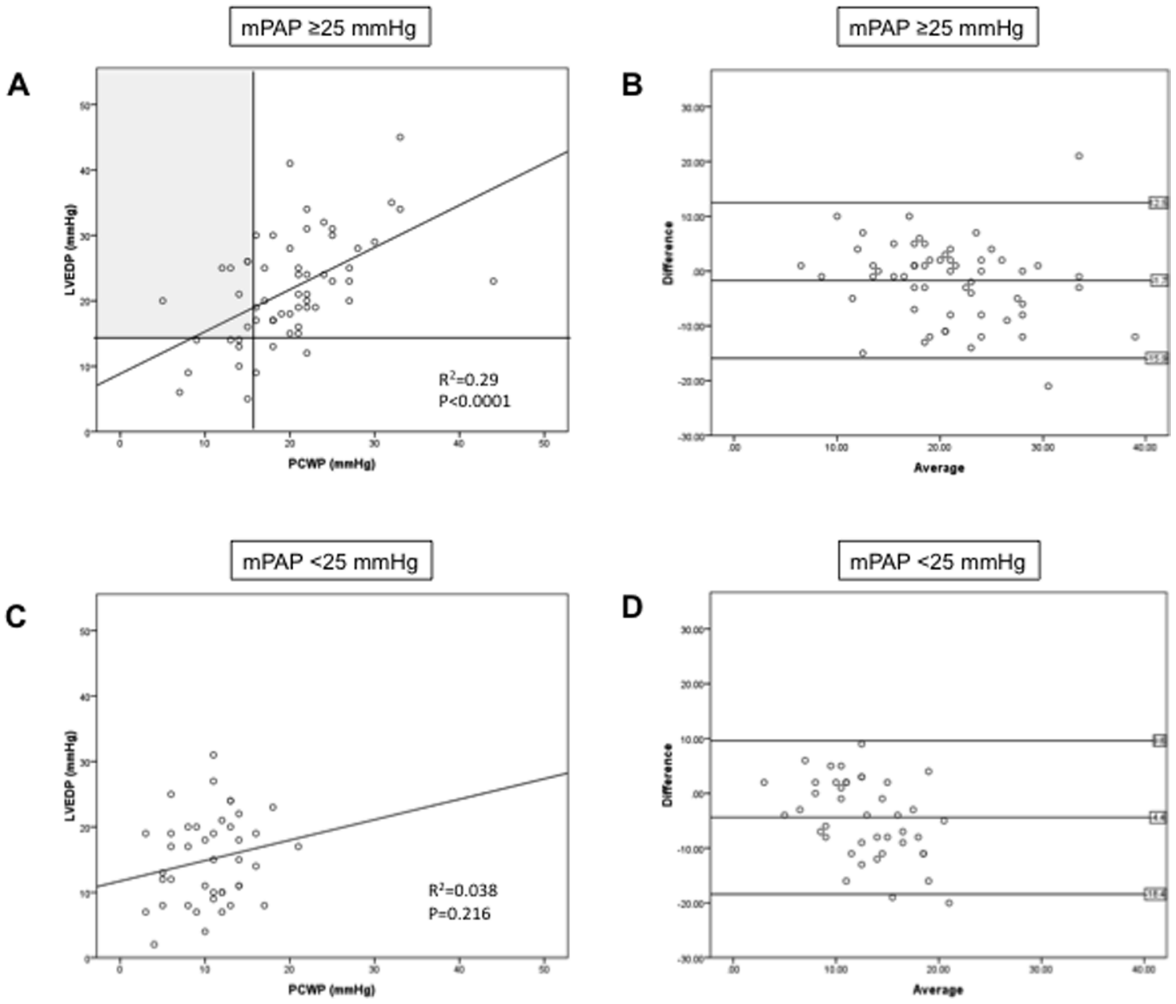
Correlation and agreement between PCWP and LVEDP in patients with (A, B) or without (C, D) pulmonary hypertension. Vertical line in (A) divides patients in patients with PCWP ≤15 mmHg or >15 mmHg; horizontal line divides patients in patients with LVEDP ≤15 mmHg or >15 mmHg. Shaded area in (A) represents the patients with PCWP ≤15 mmHg, but LVEDP >15 mmHg, thus indicating patients that would have been incorrectly classified as pre-capillary PH in absence of LVEDP measurement. See fig. 3 for explanation of Bland-Altman plot labeling.

Of the 59 patients with PH, 44 (74.6%) met PCWP criteria for PVH (Fig. 1; fig. 4A). On the other hand, based on PCWP ≤15 mmHg, 15 patients (26.4%) met criteria for pre-capillary PH (Fig. 1; fig. 4A). However, 7 of these 15 patients (46.7%) would be classified as PVH if LVEDP was used instead of PCWP (Fig. 1; shaded area in fig. 4A). On the other hand, among the 44 patients diagnosed with PVH based on elevated PCWP, only 5 individuals (11.4%) would meet pre-capillary PH criteria if LVEDP was used instead.

When limiting our correlation analysis to PH patients, a moderate correlation was confirmed (r = 0.539, r^2^ = 0.290; Fig. 4A). PCWP underestimated LVEDP by 1.7 mmHg (95%CI −3.64 to 0.15); 95% limits of agreement were −15.94 to 12.54 mmHg (Fig. 4B). On the other hand, when investigating the 42 patients without PH, no significant PCWP-LVEDP correlation was found (Fig. 4C). PCWP underestimated LVEDP by 4.4 mmHg (95%CI −6.68 to −2.21); 95% limits of agreement were −18.45 to 9.6 mmHg (Fig. 4D). These data suggest that correlation and agreement between PCWP and LVEDP are even less robust in patients without PH.

### PCWP performance in patients with increased PVR, TPG or DPG

**Figure 5:**
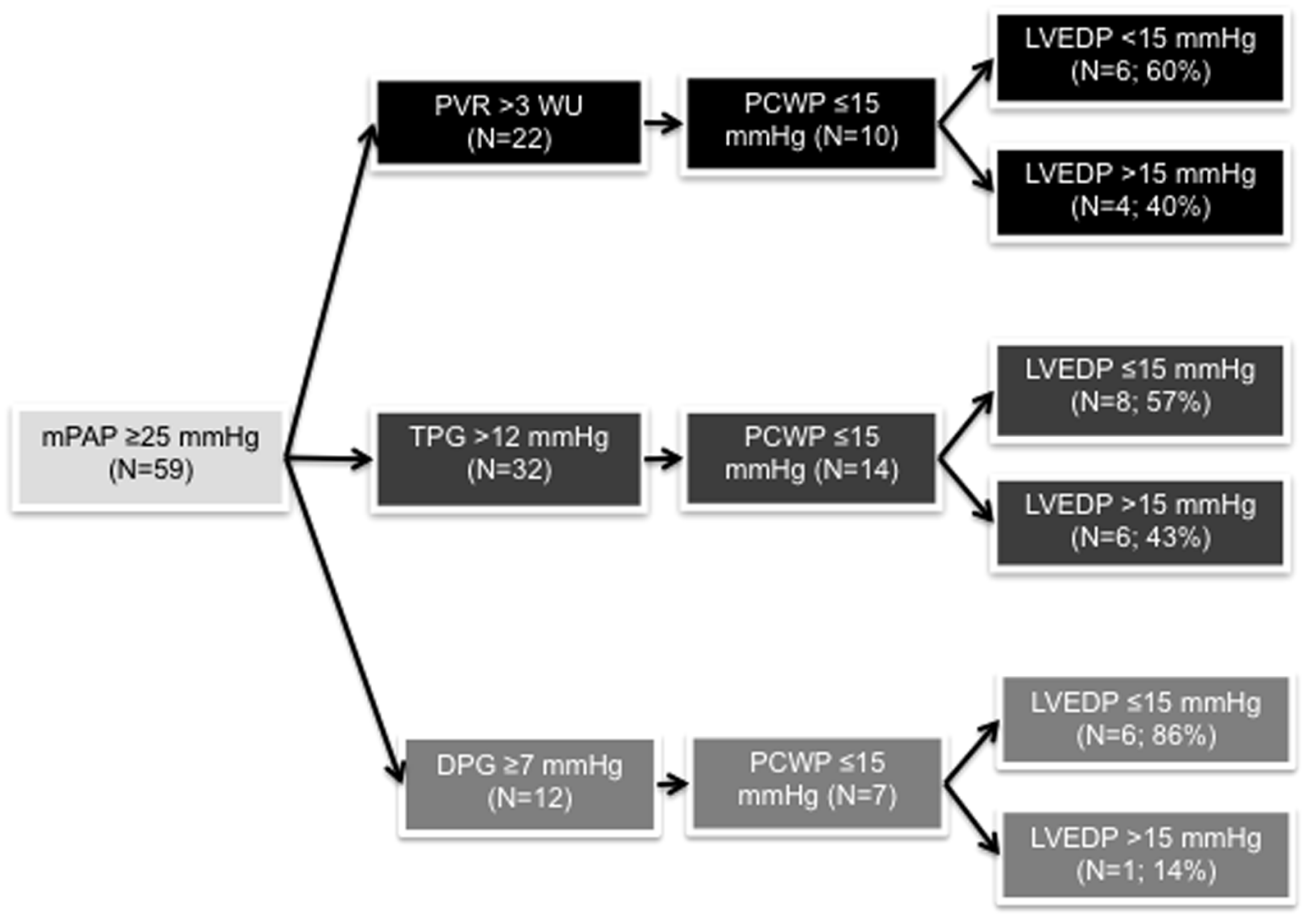
Distribution of patients with LVEDP ≤15 mmHg or LVEDP >15 mmHg in patients with PCWP ≤15 mmHg and PVR >3 WU (upper panel; black), TPG >12 mmHg (middle panel; dark grey), or DPG >7 mmHg (bottom panel; light grey). DPG, diastolic pressure gradient; PVR, pulmonary vascular resistance; TPG, transpulmonary gradient.

We then restricted our analysis to patients with PVR >3 WU, as this indicates significant pulmonary vascular remodeling and/or decreased CO[1], [16], [17]. PVR >3 WU was present in 22 patients (Fig. 5), all of which would be classified as having PH based on mPAP ≥25 mmHg. Ten patients (45.5%) would be classified as having pre-capillary PH based on PCWP ≤15 mmHg. However, if LVEDP >15 mmHg was used instead of PCWP, 4 of those 10 patients (40%) would be reclassified as having PVH. Among the 12 patients classified as having PVH based on PCWP >15 mmHg, only one (8.3%) would be reclassified as having pre-capillary PH if LVEDP was used instead.

A similar pattern was observed when analyzing the 38 patients with TPG >12 mmHg. Thirty-two of those had an mPAP ≥25 mmHg (Fig. 5). Fourteen (43%) of these patients would be categorized as pre-capillary PH based on PCWP ≤15 mmHg. However, 6 (42%) of those 14 patients would actually be categorized as PVH based on LVEDP >15 mmHg. Among the 18 patients categorized as PVH based on PCWP >15 mmHg, only 1 patient (5%) would be reclassified as pre-capillary PH if LVEDP was used instead.

Conversely, among the 12 PH patients with DPG ≥7 mmHg, 7 had a PCWP ≤15 mmHg, and only 1 (14%) of these patients was re-classified as PVH based on LVEDP >15 mmHg (Fig. 5). This suggests that in presence of a DPG ≥7, the diagnostic accuracy of a PCWP ≤15 mmHg may be better than in patients with increased TPG or PVR.

### AUROC analyses

**Figure 6:**
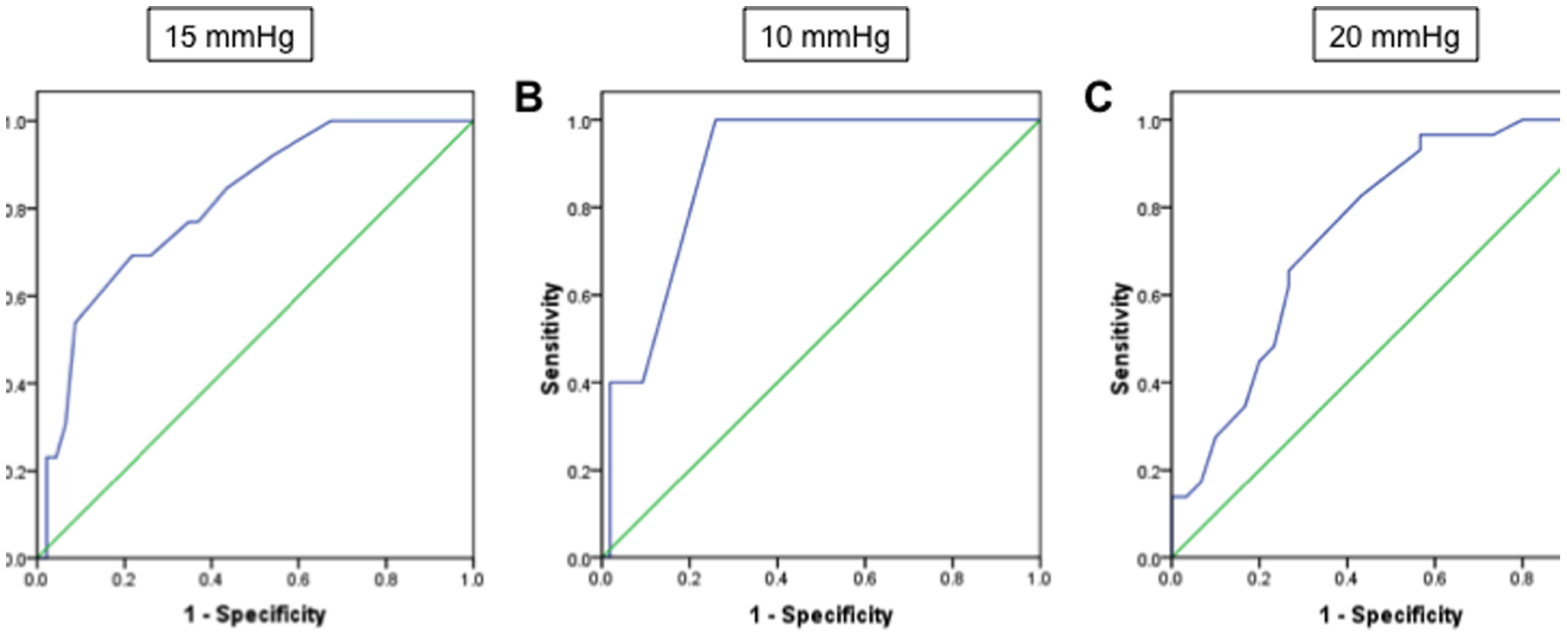
Area under the receiver operating characteristic curve (AUROC) of PCWP against LVEDP in the entire study population. (A) AUROC  =  0.81 (95% CI, 0.69 to 0.94) using a cut point of LVEDP ≤15 mmHg to indicate pre-capillary PH. (B) If a cut point of LVEDP ≤10 mmHg was used, the AUROC would be 0.89 (95% CI, 0.79 to 0.99). (C) If a cut point of LVEDP ≤20 mmHg was used, the AUROC would be 0.75 (95% CI, 0.63 to 0.88). Sensitivity = sensitivity for the outcome of LVEDP >15 mmHg; Specificity = specificity for the outcome of LVEDP >15 mmHg.

Among the 59 patients with PH, AUROC of PCWP against LVEDP was 0.81 (95%CI 0.69–0.94) when using a cutpoint of LVEDP ≤15 mmHg (Fig. 6A). This indicates that among all randomly selected pairs of patients in which one has an LVEDP ≤15 mmHg and the other has an LVEDP >15 mmHg, the patients with the higher LVEDP would have the higher PCWP in 81% of cases. If an LVEDP cutpoint of 10 or 20 mmHg was used, AUROC was 0.89 (95%CI, 0.79–0.99) and 0.75 (95%CI, 0.63–0.88) respectively (Fig. 6B+C). For the 42 patients without PH, 17 out of 37 patients with a PCWP ≤15 mmHg had an LVEDP >15 mmHg. AUROC analysis of PCWP against LVEDP among these patients was able to discriminate patients with high or low LVEDP in 76% (95%CI, 0.67–0.86; data not shown).

### PCWP and LVEDP correlations according to age, body weight, and comorbidities

**Figure 7:**
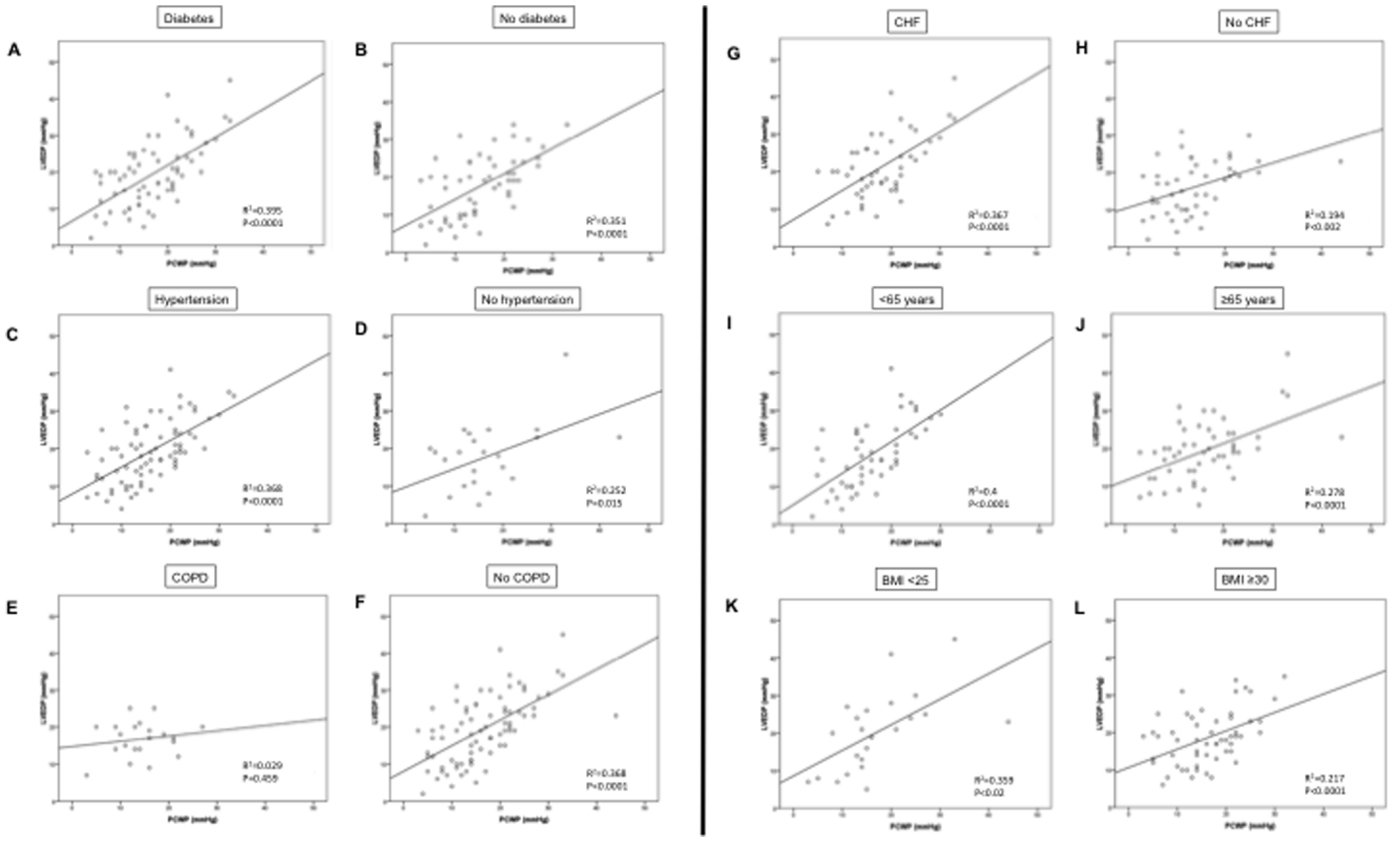
Correlations between PCWP and LEDP in patients with or without diabetes (A, B), hypertension (C, D), COPD (E, F) or CHF (G, H), as well as for patients < or ≥65 years of age (I, J), and for patients with a body mass index (BMI) <25 or ≥30 (K, L). Note lack of significant correlation between PCWP and LVEDP in COPD patients, and poor correlation in obese patients.

Lastly, we investigated correlations between PCWP and LVEDP in presence and absence of clinically important patient characteristics (Fig. 7). We detected the strongest correlations between PCWP and LVEDP in patients with diabetes and in patients <65 years of age (r = 0.62, r^2^ = 0.395, and r = 0.63, r^2^ = 0.4, respectively; p<0.0001 for both), while no significant PCWP-LVEDP correlation was found in COPD patients (r = 0.17, p = 0.46). While a significant PCWP-LVEDP correlation was found in obese patients, this correlation was weak, and the correlation coefficient was much lower than the one in patients with a BMI <25.

## Discussion

This is the first study to evaluate the PCWP-LVEDP relationship in veterans, a unique patient population with male predominance, advanced age, and multiple cardiopulmonary comorbidities [9]. Since cardiopulmonary hemodynamics are affected by age and gender [10]–[12], specific studies in this population are needed; results from other cohorts cannot necessarily be extrapolated to veterans.

In this cohort of veteran patients referred for RHC and LHC, PCWP and LVEDP are moderately correlated and poorly calibrated. PCWP tends to underestimate LVEDP by almost 3 mmHg, but may underestimate by as much as 17 mmHg, and overestimate by as much as 11 mmHg. Excluding patients with severe aortic stenosis did not significantly alter this relationship. Importantly, 46.7% of PH patients would be classified as having pre-capillary PH if the PCWP was used instead of LVEDP (a common clinical practice). LVEDP measurements, however, indicate that these patients have PVH rather than pre-capillary PH. This has clinical relevance, since pulmonary vasodilators indicated for pre-capillary PH may worsen heart failure and increase mortality in PVH [4]–[7]. This is of importance in this veteran population characterized by a high prevalence of cardiovascular and pulmonary comorbidities, and by poor cardiopulmonary reserve. These patients may not tolerate the pulmonary flooding and increased LV preload caused by pulmonary vasodilators in the setting of LV disease [4], [5]. Furthermore, in light of the high cost of PAH-specific therapy, it is important that such therapy is administered to the correct patient population.

The use of PCWP as a surrogate for LVEDP is based on multiple clinical assumptions [17]. Most importantly, it assumes a static blood column between the balloon of the PA catheter and the LV. However, this assumption may be violated in the setting of mitral valve stenosis, high alveolar pressures (as can be seen with mechanical ventilation or air trapping), significant tachycardia or hypovolemia, and if the tip of the PA catheter is not exposed to West zone 3 conditions. While mitral stenosis was an exclusion criterion in our study, it is possible that tachyarrhythmias, hypovolemia, and/or non-West zone 3 conditions may have contributed to the discrepancies between PCWP and LVEDP. Furthermore, errors in measurement and interpretation of hemodynamic values may have contributed. Lastly, use of machine-generated (digitized) mean PCWP readings instead of manually determined end-expiratory values results in underestimation of LVEDP [13]. Discrepancies are particularly pronounced in patients with increased work of breathing, a condition frequently observed in cardiopulmonary disease or obesity [16]. We used digitized mean PCWP readings, and it therefore comes as no surprise that the PCWP-LVEDP correlation was particularly poor in patients with COPD. Air trapping from COPD also may have affected the PCWP-LVEDP relationship. Along those lines, even though we found a correlation between PCWP and LVEDP in obese patients, this correlation was weak, and much less robust than the one observed in patients with a normal BMI. These data confirm that PCWP values are particularly prone to error in patients with significant respiratory excursions (as can be seen in COPD or obesity).

Almost 60% percent of our patients had PH. This is significantly higher than the 37.5% of patients with PH in the study by Halpern and Taichman [8]. Similarly, 22% of our patients had a PVR >3 WU, compared to roughly 10% in the Halpern study. As evident from table 1, there was a high prevalence of chronic heart and lung disease, sleep-disordered breathing and obesity, all of which may cause PH [18], [19]. This may explain the higher incidence of PH in our population. Despite the higher prevalence of PH in the current study, and despite differences in patient characteristics, the calibration of PCWP and LVEDP was identical in both studies (mean bias −2.9 mmHg). As in the Halpern/Taichman study, in the absence of LVEDP measurement almost 50% of patients would be misclassified in our study as pre-capillary PH instead of PVH. These data suggest that the PCWP-LVEDP relationship is surprisingly robust across different patient populations.

It is noteworthy that correlation and agreement between PCWP and LVEDP were worse in the group of patients without PH, where PCWP also tended to underestimate LVEDP. While less important to PH diagnosis and classification, this is of relevance to situations in which a patient's volume status or cardiac function needs to be determined. In fact, underestimation of LVEDP by PCWP also occurs in patients with acute myocardial infarction [20] and in critically ill patients [21]. This poor calibration may explain at least in part why the majority of studies of pulmonary artery catheter-guided fluid therapy have been disappointing [22], [23].

As discussed by others [8], simply adding the mean bias to the PCWP is not sufficient, since the wide 95% limits of agreement demonstrate that - even though PCWP tends to underestimate LVEDP - there is a significant number of patients in which PCWP *overestimates* the latter. Systemic bias therefore is not the only explanation for the discrepancy between PCWP and LVEDP, and for any given patient it is impossible to predict whether the PCWP is higher or lower than the LVEDP. If accurate determination of cardiac filling pressures is needed, threshold for LVEDP measurement should be low.

Our results have important implications for veterans. First, PVH is common in this population (78% of all PH patients had LVEDP-confirmed PVH). This condition therefore should be aggressively looked for. We do not propose, however, aggressive treatment of PVH with PAH-specific therapy. Rather, if PVH is diagnosed, the patient should be aggressively treated with proven pharmacologic and non-pharmacologic (e.g. exercise, oxygen for hypoxemia, CPAP for obstructive sleep apnea) interventions [24].

Second, providers need to be cognizant about the potential for underdiagnosis (and, to a lesser extent, overdiagnosis) of PVH if decisions are based solely on PCWP. As previously shown [13], [16], software-generated mean PCWPs frequently are inaccurate and should not be relied upon. However, even with end-expiratory PCWP there still is potential for discrepancies between PCWP and LVEDP (95% limits of agreement −5.19 to 6.93 mmHg[13]). When an accurate assessment of volume status or LV function is needed, determination of LVEDP should strongly be considered (especially if the pre-test probability for PVH is high). We consider the additional risks of LVEDP determination small as compared to the risks of inappropriate pulmonary vasodilator therapy in the setting of occult LV disease. An alternative strategy to detect occult LV disease is provocative testing during RHC (e.g. volume challenge or exercise testing) [16]. However, while promising, these provocative maneuvers still need prospective evaluation.

Misclassification of PVH as pre-capillary PH in our cohort appears to be less common in the subgroup of patients with a DPG of ≥7 mmHg. While this observation is limited by a relatively small number of patients, it is consistent with the recently reported superior ability of the DPG to recognize significant PH as compared to the TPG [15].

The major strength of our study is the investigation of a unique and clinically important patient population. Furthermore, since prior work did not investigate patient characteristics other than PH (e.g. age, BMI, co-morbidities), the investigation of these factors on the PCWP-LVEDP relationship represents another specific strength of our study.

Our study has limitations. First, this is a retrospective single center study with a limited number of patients. Our cohort is identical in age and sex distribution to other recently VA populations [9]. Similarly, the predominance of elderly Caucasian males is similar to what is observed at other large VA medical centers in the US (G. Choudhary, B. Maron; personal communication). However, despite these typical characteristics of VA cohort, our results are similar to those from a larger population [8], thus supporting their validity and generalizability. Second, selection bias may have resulted from referral of patients for combined RHC/LHC. However, most patients (>80%) were actually referred for RHC or LHC only, and the indication for combined catheterization was made later by the procedural cardiologist based on patient characteristics or intra-procedural findings in order to better understand the clinical scenario. Referral for combined RHC/LHC occurred in <20% of cases. Furthermore, previous studies demonstrated no differences in PCWP between patients undergoing combined RHC/LHC and those undergoing RHC alone. Third, hemodynamic tracings were not stored. We therefore were not able to compare the digitized with end-expiratory PCWPs. This will be the subject of further prospective study.

In conclusion, in veterans undergoing combined RHC and LHC, PCWP was poorly calibrated to LVEDP. Reliance on PCWP rather than LVEDP misclassifies veterans as having pre-capillary PH rather than PVH in almost 50% of cases. Correlations were particularly poor in patients with COPD and obesity, suggesting a significant impact of respiratory excursions on the PCWP-LVEDP relationship if automated PCWP readings are used. This is clinically relevant, as misclassification may lead to inappropriate therapies and adverse events, a particular problem in this population with high incidence of cardiovascular comorbidities and poor cardiac reserve.

## References

[1] McLaughlin VV, Archer SL, Badesch DB, Barst RJ, Farber HW, et al (2009) ACCF/AHA 2009 expert consensus document on pulmonary hypertension a report of the American College of Cardiology Foundation Task Force on Expert Consensus Documents and the American Heart Association developed in collaboration with the American College of Chest Physicians; American Thoracic Society, Inc.; and the Pulmonary Hypertension Association. J Am Coll Cardiol 53: 1573–1619. 19389575 10.1016/j.jacc.2009.01.004

[2] Galie N, Hoeper MM, Humbert M, Torbicki A, Vachiery JL, et al (2009) Guidelines for the diagnosis and treatment of pulmonary hypertension. Eur Respir J 34: 1219–1263. 19749199 10.1183/09031936.00139009

[3] Badesch DB, Champion HC, Sanchez MA, Hoeper MM, Loyd JE, et al (2009) Diagnosis and assessment of pulmonary arterial hypertension. J Am Coll Cardiol 54: S55–66. 19555859 10.1016/j.jacc.2009.04.011

[4] Hoeper MM, Barbera JA, Channick RN, Hassoun PM, Lang IM, et al (2009) Diagnosis, assessment, and treatment of non-pulmonary arterial hypertension pulmonary hypertension. J Am Coll Cardiol 54: S85–96. 19555862 10.1016/j.jacc.2009.04.008

[5] Guazzi M, Borlaug BA (2012) Pulmonary hypertension due to left heart disease. Circulation 126: 975–990. 22908015 10.1161/CIRCULATIONAHA.111.085761

[6] Califf RM, Adams KF, McKenna WJ, Gheorghiade M, Uretsky BF, et al (1997) A randomized controlled trial of epoprostenol therapy for severe congestive heart failure: The Flolan International Randomized Survival Trial (FIRST). Am Heart J 134: 44–54. 9266782 10.1016/s0002-8703(97)70105-4

[7] Packer M, McMurray J, Massie BM, Caspi A, Charlon V, et al (2005) Clinical effects of endothelin receptor antagonism with bosentan in patients with severe chronic heart failure: results of a pilot study. J Card Fail 11: 12–20. 15704058 10.1016/j.cardfail.2004.05.006

[8] Halpern SD, Taichman DB (2009) Misclassification of pulmonary hypertension due to reliance on pulmonary capillary wedge pressure rather than left ventricular end-diastolic pressure. Chest 136: 37–43. 19255293 10.1378/chest.08-2784

[9] Kaboli PJ, Go JT, Hockenberry J, Glasgow JM, Johnson SR, et al (2012) Associations between reduced hospital length of stay and 30-day readmission rate and mortality: 14-year experience in 129 Veterans Affairs hospitals. Ann Intern Med 157: 837–845. 23247937 10.7326/0003-4819-157-12-201212180-00003

[10] Kovacs G, Berghold A, Scheidl S, Olschewski H (2009) Pulmonary arterial pressure during rest and exercise in healthy subjects: a systematic review. Eur Respir J 34: 888–894. 19324955 10.1183/09031936.00145608

[11] Shapiro S, Traiger GL, Turner M, McGoon MD, Wason P, et al (2012) Sex differences in the diagnosis, treatment, and outcome of patients with pulmonary arterial hypertension enrolled in the registry to evaluate early and long-term pulmonary arterial hypertension disease management. Chest 141: 363–373. 21757572 10.1378/chest.10-3114

[12] Kawut SM, Lima JA, Barr RG, Chahal H, Jain A, et al (2011) Sex and race differences in right ventricular structure and function: the multi-ethnic study of atherosclerosis-right ventricle study. Circulation 123: 2542–2551. 21646505 10.1161/CIRCULATIONAHA.110.985515 PMC3111939

[13] Ryan JJ, Rich JD, Thiruvoipati T, Swamy R, Kim GH, et al (2012) Current practice for determining pulmonary capillary wedge pressure predisposes to serious errors in the classification of patients with pulmonary hypertension. Am Heart J 163: 589–594. 22520524 10.1016/j.ahj.2012.01.024

[14] Maron BA, Choudhary G, Khan UA, Jankowich MD, McChesney H, et al (2013) Clinical profile and underdiagnosis of pulmonary hypertension in US veteran patients. Circ Heart Fail 6: 906–912. 23811965 10.1161/CIRCHEARTFAILURE.112.000091 PMC3894604

[15] Gerges C, Gerges M, Lang MB, Zhang Y, Jakowitsch J, et al (2013) Diastolic pulmonary vascular pressure gradient: a predictor of prognosis in “out-of-proportion” pulmonary hypertension. Chest 143: 758–766. 23580984 10.1378/chest.12-1653

[16] Champion HC, Michelakis ED, Hassoun PM (2009) Comprehensive invasive and noninvasive approach to the right ventricle-pulmonary circulation unit: state of the art and clinical and research implications. Circulation 120: 992–1007. 19752350 10.1161/CIRCULATIONAHA.106.674028

[17] Sharkey SW (1997). A Guide to Interpretation of Hemodynamic Data in the Coronary Care Unit. 1st edition. Philadelphia, PA: Lippincott Williams and Wilkins.

[18] Rabinovitch M (2012) Molecular pathogenesis of pulmonary arterial hypertension. J Clin Invest 122: 4306–4313. 23202738 10.1172/JCI60658 PMC3533531

[19] Morrell NW, Adnot S, Archer SL, Dupuis J, Jones PL, et al (2009) Cellular and molecular basis of pulmonary arterial hypertension. J Am Coll Cardiol 54: S20–31. 19555855 10.1016/j.jacc.2009.04.018 PMC2790324

[20] Rahimtoola SH, Loeb HS, Ehsani A, Sinno MZ, Chuquimia R, et al (1972) Relationship of pulmonary artery to left ventricular diastolic pressures in acute myocardial infarction. Circulation 46: 283–290. 5046023 10.1161/01.cir.46.2.283

[21] Calvin JE, Driedger AA, Sibbald WJ (1981) Does the pulmonary capillary wedge pressure predict left ventricular preload in critically ill patients? Crit Care Med 9: 437–443. 7226863 10.1097/00003246-198106000-00001

[22] Wheeler AP, Bernard GR, Thompson BT, Schoenfeld D, Wiedemann HP, et al (2006) Pulmonary-artery versus central venous catheter to guide treatment of acute lung injury. N Engl J Med 354: 2213–2224. 16714768 10.1056/NEJMoa061895

[23] Shure D (2006) Pulmonary-artery catheters–peace at last? N Engl J Med 354: 2273–2274. 16714770 10.1056/NEJMe068099

[24] Hunt SA, Abraham WT, Chin MH, Feldman AM, Francis GS, et al (2009) 2009 focused update incorporated into the ACC/AHA 2005 Guidelines for the Diagnosis and Management of Heart Failure in Adults: a report of the American College of Cardiology Foundation/American Heart Association Task Force on Practice Guidelines: developed in collaboration with the International Society for Heart and Lung Transplantation. Circulation 119: e391–479. 19324966 10.1161/CIRCULATIONAHA.109.192065

